# Protective Effects of Pasireotide in LPS-Induced Acute Lung Injury

**DOI:** 10.3390/ph18070942

**Published:** 2025-06-22

**Authors:** Saikat Fakir, Md Matiur Rahman Sarker, Madan Sigdel, Nektarios Barabutis

**Affiliations:** School of Basic Pharmaceutical and Toxicological Sciences, College of Pharmacy, University of Louisiana Monroe, Monroe, LA 71201, USA; fakirs@warhawks.ulm.edu (S.F.); sarkermm@warhawks.ulm.edu (M.M.R.S.); sigdelm@warhawks.ulm.edu (M.S.)

**Keywords:** Somatostatin, inflammation, homeostasis, sepsis, growth hormone

## Abstract

**Background/Objectives:** Acute lung injury (ALI) is an inflammatory condition characterized by tissue barrier damage, which leads to vascular leakage, pulmonary edema, and compromised gas exchange. Lipopolysaccharides (LPS) are a component of Gram-negative bacteria, which trigger inflammation by Toll-like receptor 4 (TLR4) activation. Herein, we investigated the possibility that Pasireotide (PAS) exerts protective effects in an experimental model of ALI. **Methods:** C57BL/6 male mice received an intratracheal injection of saline or LPS, followed by PAS or vehicle treatment. Bronchoalveolar lavage fluid (BALF) was collected via tracheal catheterization, and Western blot analysis was used to detect protein expression variations. **Results:** Our results suggest that PAS treatment alleviates LPS-induced mouse lung injury and inflammation. JAK/STAT and MAPK activation levels in the inflamed lungs were suppressed due to PAS treatment, as well as BALF protein concentration. Additionally, PAS counteracted LPS-induced Grp94 protein reduction, suggesting the involvement of ATF6 in PAS-triggered barrier-protective effects. Grp94 is a downstream ATF6 target. **Conclusions:** Our data demonstrate that PAS protects mouse lungs against LPS in an experimental model of ALI.

## 1. Introduction

Inflammation is a biological response against harmful stimuli (e.g., pathogens or toxins) or tissue injury, which aims to maintain homeostasis by initiating repair processes [1,2]. Chronic or excessive inflammation [3] can contribute to disease pathogenesis involving organ damage, increased vascular permeability, and impaired lung/gas exchange [4,5,6].

Excessive lung inflammation is a hallmark of respiratory disorders (e.g., asthma and COPD) and involves persistent immune activation and tissue remodeling [7,8]. Acute lung injury (ALI), a life-threatening disorder, is characterized by alveolar damage, leukocyte infiltration, and disruption of the alveolar–capillary barrier [9]. Our better understanding of inflammatory lung disease may contribute to preventing the transition from protective immune responses to pathological conditions [10].

Lipopolysaccharides (LPS), a major component of the outer membrane of Gram-negative bacteria, trigger inflammatory responses by activating Toll-like receptor 4 (TLR4) [11,12]. The aforementioned activities lead to the recruitment of neutrophils into the lungs, which in turn results in cytokine, chemokine, and proteolytic enzyme release, compromising epithelial and endothelial integrity [13]. Consequently, protein-rich fluid accumulates in the alveolar spaces, impairing oxygenation [14]. More effective pharmacological treatments that ameliorate lung injury are needed, as evident by the high mortality rates associated with sepsis and ARDS.

Somatostatin (SST) is a peptide hormone that inhibits growth hormone secretion [15], pro-inflammatory cytokine release, and immune cell activation [16]. Somatostatin receptor (SSTR) signaling exerts potent anti-inflammatory effects by inhibiting pro-inflammatory cytokine release, immune cell activation, and vascular hyperpermeability [17,18,19,20,21,22]. Pasireotide (PAS) is a second-generation multi-receptor synthetic somatostatin analog (SSA), which is widely used in clinics for Cushing’s syndrome and acromegaly treatment [23,24,25]. An emerging body of evidence suggests that long-acting SSAs exhibit anti-inflammatory properties [26,27], but the available information on the corresponding field is limited. Interestingly, it was recently revealed that PAS exerts anti-inflammatory effects in endothelial cells in vitro [28].

In the present study, we examined the potential therapeutic potential of PAS in an established murine model of LPS-induced ALI. Mice exposed intratracheally to LPS were post-treated with PAS to examine the possibility that this compound can ameliorate endothelial injury and inflammation. Our observations suggest that PAS mitigates lung damage caused by LPS, suggesting the possibility that it could be repurposed toward disorders related to barrier dysfunction.

## 2. Results

### 2.1. PAS Reduces BALF Protein Concentration Levels in Mice Treated with LPSs

**Figure 1:**
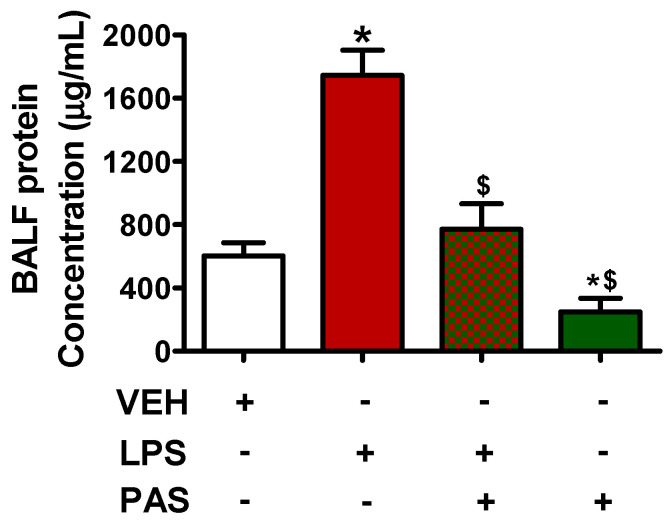
C57BL/6 mice were treated with an intratracheal (IT) injection of saline or LPS (1.6 mg/kg, dissolved in saline) and were post-treated (24 h after LPS) with a subcutaneous injection of either a vehicle (0.1% DMSO in 10% 1,2-propanediol) or PAS (5 mg/kg, dissolved in 0.1% DMSO and 10% 1,2-propanediol) once daily for 3 days. After treatment, lung BALF was obtained by instilling and withdrawing 1 mL of PBS using a tracheal cannula. * *p* < 0.05 vs. vehicle (VEH); ^$^ *p* < 0.05 vs. LPS. The graph represents 6 animals per group. Mean ± SEM.

C57BL/6 mice were treated intratracheally with LPS (1.6 mg/kg) or saline for 24 h and were then subjected to daily subcutaneous injections of vehicle or Pasireotide (5 mg/kg) for 3 days (one injection per day). After treatment, BALF was collected, and protein concentration was measured, per the procedures described in the Materials and Methods Section. Our observations suggest that PAS treatment counteracted LPS-induced BALF protein concentration increase (Figure 1), hence mitigating lung edema.

### 2.2. PAS Inhibits LPS-Induced Activation of the JAK/STAT Signaling Pathway in Mouse Lungs

**Figure 2:**
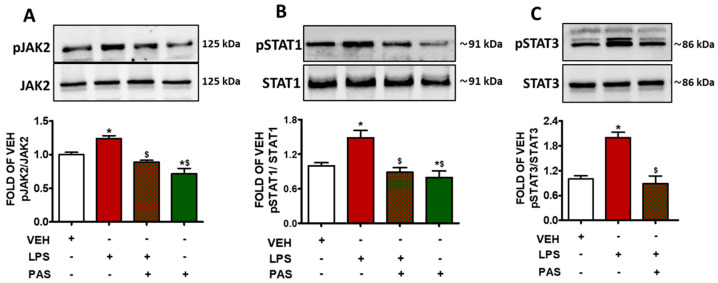
Western blot analysis of (**A**) phosphorylated JAK2 (pJAK2) and JAK2, (**B**) phosphorylated STAT1 (pSTAT1) and STAT1, (**C**) phosphorylated STAT3 (pSTAT3) and STAT3 in lung lysates of C57BL/6 mice that were treated with a vehicle (saline) or LPS (1.6 mg/kg, IT) for 24 h prior to vehicle (0.1% DMSO in 10% 1,2-propanediol) or 5 mg/kg PAS (once daily for 3 days, dissolved in 0.1% DMSO, 10% 1,2-propanediol, SC) treatment. Signal intensity was analyzed by densitometry. The pJAK2, pSTAT1, and pSTAT3 protein expression levels were normalized to JAK2, STAT1, and STAT3, respectively. * *p* < 0.05 vs. VEH; ^$^ *p* < 0.05 vs. LPS; *n* = 3. Mean ± SEM.

Mice exposed to LPS, to induce ALI, were post-treated 24 h after with PAS. LPS administration increased JAK2 (pJAK2) phosphorylation (Figure 2A), whereas GH inhibition due to PAS substantially suppressed JAK2 activation. Similar effects were observed in the case of pSTAT1 (Figure 2B) and pSTAT3 (Figure 2C). Both phosphorylated protein levels were elevated due to LPS exposure, and those effects were counteracted by PAS post-treatment. Collectively, our results suggest that PAS ameliorates lung inflammation—at least in part—via JAK2/STAT1/STAT3 suppression.

### 2.3. PAS Attenuates LPS-Induced MAPK Activation in Mouse Lungs

**Figure 3:**
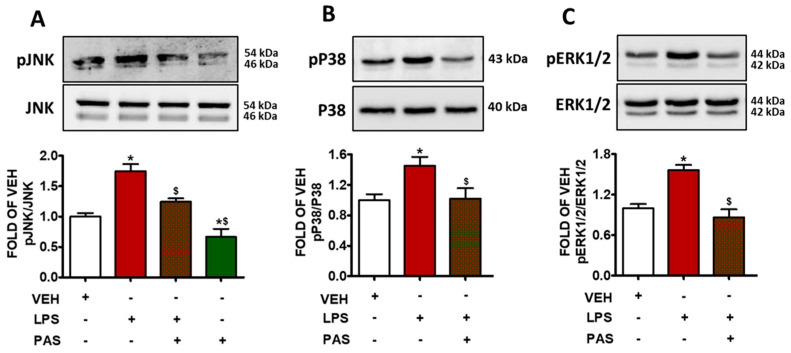
Western blot analysis of (**A**) phosphorylated JNK (pJNK) and JNK, (**B**) phosphorylated P38 (pP38) and P38, (**C**) phosphorylated ERK1/2 (pERK1/2) and ERK1/2 in lung lysates of C57BL/6 mice that were treated with a vehicle (saline) or LPS (1.6 mg/kg, IT) for 24 h prior to vehicle (0.1% DMSO in 10% 1,2-propanediol) or 5 mg/kg PAS (once daily for 3 days, dissolved in 0.1% DMSO, 10% 1,2-propanediol, SC) treatment. Signal intensity was analyzed by densitometry, and pJNK, pP38, and pERK1/2 protein expression levels were normalized to JNK, P38, and ERK1/2, respectively. * *p* < 0.05 vs. VEH; ^$^ *p* < 0.05 vs. LPS; *n* = 3. Mean ± SEM.

The exposure of mouse lungs to LPS increased the levels of phosphorylated JNK (pJNK) (Figure 3A), phosphorylated P38 (pP38) (Figure 3B), and phosphorylated ERK1/2 (pERK1/2) (Figure 3C). The activation of those kinases was significantly suppressed due to PAS post-treatment; hence, it is suggested that PAS inhibits LPS-induced MAPK activation, which was previously shown to mediate inflammatory responses.

### 2.4. PAS Counteracts LPS-Induced Grp94 Suppression in Inflamed Mouse Lungs

**Figure 4:**
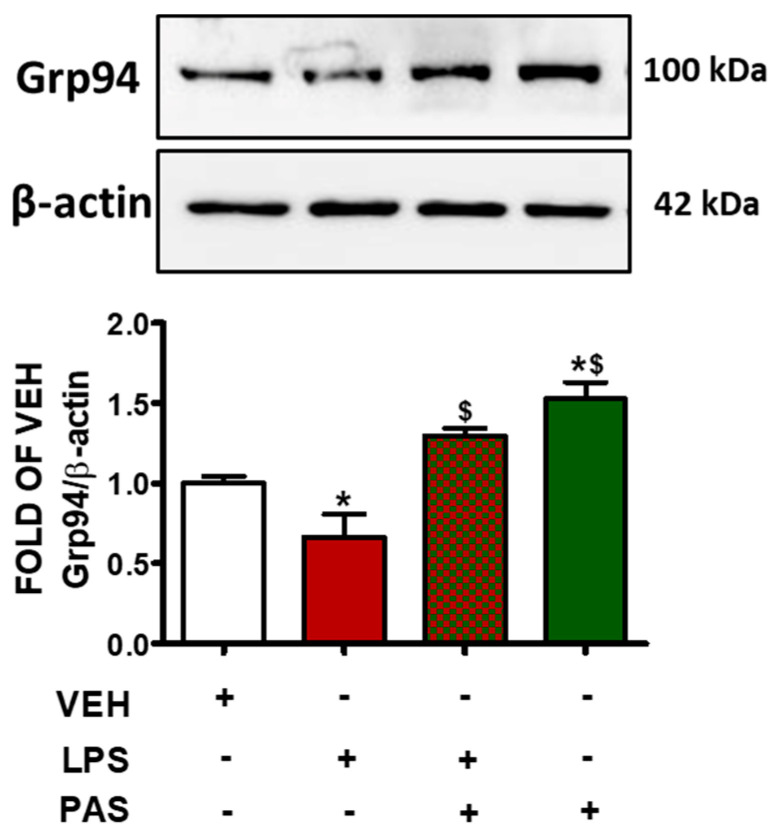
Western blot analysis of Grp94 in lung lysates of C57BL/6 mice that were treated with a vehicle (saline) or LPS (1.6 mg/kg, IT) for 24 h prior to vehicle (0.1% DMSO in 10% 1,2-propanediol) or 5 mg/kg PAS (once daily for 3 days, dissolved in 0.1% DMSO, 10% 1,2-propanediol, SC) treatment. Signal intensity was analyzed by densitometry. Protein levels of Grp94 were normalized to β-actin. The blots shown are representative of three independent experiments. * *p* < 0.05 vs. VEH and ^$^ *p* < 0.05 vs. LPS. Mean ± SEM.

In mouse lungs, LPS administration suppressed Grp94 protein expression levels (Figure 4). Those effects were opposed by PAS post-treatment. This is important because Grp94 is a major ER-resident chaperone involved in protein folding, cell stress response, and cellular homeostasis, and indicates barrier-protective activities via ATF6 activation [29]. The findings indicate that PAS exerts protective effects in ALI, at least in part, by counteracting LPS-induced Grp94 suppression.

## 3. Discussion

SSAs exhibit immunomodulatory [30] and anti-inflammatory [31] activities via GH suppression [32], and they lower the expression levels of the pro-inflammatory mediator TNF-α [33] via binding to the somatostatin receptors. Octreotide (OCT)—which is a first-generation SSA—modulates cytokine production in rheumatoid arthritis synoviocytes through interleukin-15 and TNF-α inhibition and increases interleukin-10 levels [34]. Furthermore, OCT reduces BALF protein concentration in ALI [35]. The SSA Lanreotide (LAN) counteracts endothelial injury in both cells and mice [36]. Pasireotide is a synthetic cyclic hexapeptide in which the six amino acid residues—L-phenylglycine, D-tryptophan, L-lysine, O-benzylated L-tyrosine, L-phenylalanine, and a modified form of L-hydroxyproline—are covalently linked in a homodetic (amide bond only) ring structure (PubChem CID: 9941444). It binds to the somatostatin receptors (SSTRs) 1–5 [23,37], but it exhibits greater potency at adenyl cyclase inhibition through SSTR2 and SSTR5 [38].

The ALI experimental model used in the current study demonstrates hallmark features of ARDS [39] including increased vascular permeability, infiltration of inflammatory cells, and cytokine release [40,41]. Our endeavors investigate for the first time the effects of PAS on LPS-induced ALI by evaluating BALF protein concentration measurements, as well as the expression of key signaling molecules involved in inflammation and cell stress response [42]. Furthermore, Supplementary Materials are provided in support of our figures.

Our results indicate that LPS exposure significantly increases BALF total protein concentration, indicating disruption of the alveolar–capillary barrier. PAS post-treatment reduced BALF protein levels in the inflamed lungs, suggesting that this particular SSA exerts a protective role in LPS-induced ALI, which is in line with previous observations [28]. To elucidate the underlying molecular mechanisms mediating the previously described beneficial effects, we assessed STAT1, STAT3, JAK2, JNK, P38 MAPK, and ERK1/2 activation, as well as Grp94 expression, in mice exposed to PAS after LPS treatment, in a manner previously performed utilizing Hsp90 inhibitors [43]. The aforementioned proteins were studied because they are central to inflammatory injury [44,45,46,47,48]. LPS activate the JAK/STAT pathway, which in turn promotes the transcription of pro-inflammatory genes [49]. Interestingly, PAS counteracted the LPS-induced phosphorylation of JAK2, STAT1, and STAT3, suggesting that it blocks the corresponding inflammatory cascades and cytoskeletal remodeling [50].

Mitogen-activated protein kinases (MAPKs), including ERK1/2, JNK, and p38, are activated due to LPS, and they have been associated with neutrophil recruitment and barrier dysfunction [51]. JNK is known to mediate apoptosis and inflammation [52], which is similar to ERK1/2, which has been implicated in vascular regulation [53]. PAS post-treatment reduced the LPS-triggered phosphorylation of JNK, p38, and ERK1/2, suggesting that those effects are involved in PAS-related anti-inflammatory effects.

Grp94 is a stress-induced chaperone and a downstream target of ATF6 activation [54], which is a transcription factor previously involved in vascular barrier enhancement [55]. PAS increased the expression levels of Grp94 in the inflamed mouse lungs, suggesting a potential role of this SST in alleviating ER stress and inflammation via ATF6 activation. Further studies will further substantiate our observations, utilizing receptor-specific agonists or antagonists to assist in further clarifying our findings.

## 4. Materials and Methods

### 4.1. Reagents

Pasireotide Acetate (TP2207) was obtained from TargetMol, located in Wellesley, MA. Anti-rabbit IgG HRP secondary antibody (95017–556), anti-mouse IgG HRP (95017-554), nitrocellulose membranes (10063–173), DMSO (25–950-CQC), and RIPA solution (AAJ63306-AP) were purchased from VWR (Radnor, PA). Protease inhibitors (AB287909) were purchased from Abcam (Cambridge, UK). The p-p38 (9211S), p38 (9212S), pERK1/2 (9101S), ERK1/2 (9102S), pSTAT1 (9167S), STAT1 (9172S), pSTAT3 (9145S), STAT3 (4904S), p-SAPK/JNK (9251S), SAPK/JNK (9252S), p-JAK2 (3776S), JAK2 (3230S), and Grp94 (2104S) antibodies were made available from Cell Signaling Technology (Danvers, MA, USA). LPS (L4130) and β-actin antibodies (A6441) were acquired from Sigma-Aldrich (St. Louis, MO, USA).

### 4.2. In Vivo Model of ALI, PAS Treatment, and BALF Protein Concentration Measurements

Seven-week-old C57BL/6 male mice were purchased from Envigo (Indianapolis, IN, USA) and were maintained in a 12:12 h light/dark cycle. The temperature (22–24 °C) and humidity (50–60%) were carefully monitored, and all procedures were approved by the Institutional Animal Care and Use Committee (IACUC) [56]. To induce ALI, mice were treated with an intratracheal (I.T.) injection of saline or LPS (1.6 mg/kg) and were post-treated (24 h after LPS) with PAS (5 mg/kg) subcutaneously (once daily for 3 consecutive days). PAS administration dosage was selected based on our preliminary studies and previous observations, taking into account endothelial heterogeneity [57,58,59]. The bronchoalveolar lavage fluid (BALF) was collected from mice through the insertion of a catheter into the trachea, introducing PBS solution, and then aspirating the fluid to collect cells and other components from the lungs. Protein concentration was measured by the Pierce™ BCA^®^ Protein Assay.

### 4.3. Western Blot Analysis

Proteins were isolated from mouse lung tissues using RIPA buffer. Equal amounts (35 µg) of protein sample were separated by sodium dodecyl sulfate polyacrylamide gel electrophoresis. Wet transfer was used to transfer the proteins onto the nitrocellulose membranes. The blots were blocked in 5% non-fat dried milk for 60 min to prevent non-specific binding and were exposed to primary antibodies diluted in a blocking solution (1:1000) at 4 °C (16 h). Appropriate secondary antibodies (1:5000) were used to detect the target proteins. All membranes were exposed to a chemiluminescent substrate (SuperSignal West Femto (PI34096)) and developed using the ChemiDoc System from Bio-Rad (Hercules, CA, USA).

### 4.4. Densitometry and Statistical Analysis

The images were analyzed using ImageJ software (Version 1.53e) (National Institutes of Health) to quantify the intensity of protein bands from Western blot experiments. Densitometric values were obtained for each band and normalized to the corresponding loading control to correct for variations in sample loading. The normalized values were calculated for each group and expressed as mean ± standard deviation (SD). These data were then imported into GraphPad Prism software (version 5.01). For statistical analysis, a one-way analysis of variance (ANOVA) was utilized to determine whether there were significant differences in protein expression levels among the various experimental groups. Following the ANOVA, an unpaired one-tailed Student’s t-test was conducted to assess specific comparisons between individual treatment groups and the control group. A one-tailed test was selected based on the prior hypothesis that the treatments would affect protein expression. The data were entered into GraphPad Prism in a grouped format, where each column represented a different treatment condition. All statistical tests were performed with a significance threshold set at *p* < 0.05. The results were presented as mean ± SD, and statistical significance was indicated, when applicable, in the graphs.

## Data Availability

Data are available upon reasonable request.

## Supplementary Materials

The following supporting information can be downloaded at https://www.mdpi.com/article/10.3390/ph18070942/s1: Figure S1: Effects of PAS in LPS-induced JAK2, STAT1 and STAT3 phosphorylation; Figure S2: Effects of PAS in LPS-induced JNK, P38 and ERK1/2 phosphorylation; Figure S3: Effects of PAS in Grp94 expression.

## Abbreviations

The following abbreviations are used in this manuscript:

ALI: acute lung injury; COPD: chronic obstructive pulmonary disease; ARDS: acute respiratory distress syndrome; TLR4: Toll-like receptor 4; PAS: pasireotide; SST: somatostatin; SSTR: somatostatin receptor; SSA: synthetic somatostatin analogs; DMSO: dimethylsulfoxide; RIPA: radioimmunoprecipitation assay; JAK2: janus kinase 2; STAT1: signal transducer and activator of transcription 1; STAT3: signal transducer and activator of transcription 3; TNF-α: tumor necrosis factor alpha; MAPK: mitogen-activated protein kinases; BALF: bronchoalveolar lavage fluid; Grp94: glucose-regulated protein 94; JNK: c-Jun N-terminal kinase; ERK1/2: extracellular signal-regulated kinase 1/2LPS: lipopolysaccharides
