# Supplementary material for: Protective Effects of Pasireotide in LPS-Induced Acute Lung Injury
Source: Pharmaceuticals (Basel). 2025 Jun 22;18(7):942. doi: 10.3390/ph18070942 (PMC12298338; doi:10.3390/ph18070942)
Supplement: Supplementary file 1 [file pharmaceuticals-18-00942-s001.zip › pharmaceuticals-3652154-supplementary.pdf]

Figure S1: original images of Figure 2

Effects of PAS in LPS-induced JAK2, STAT1 and STAT3 phosphorylation

Figure S1 (A)

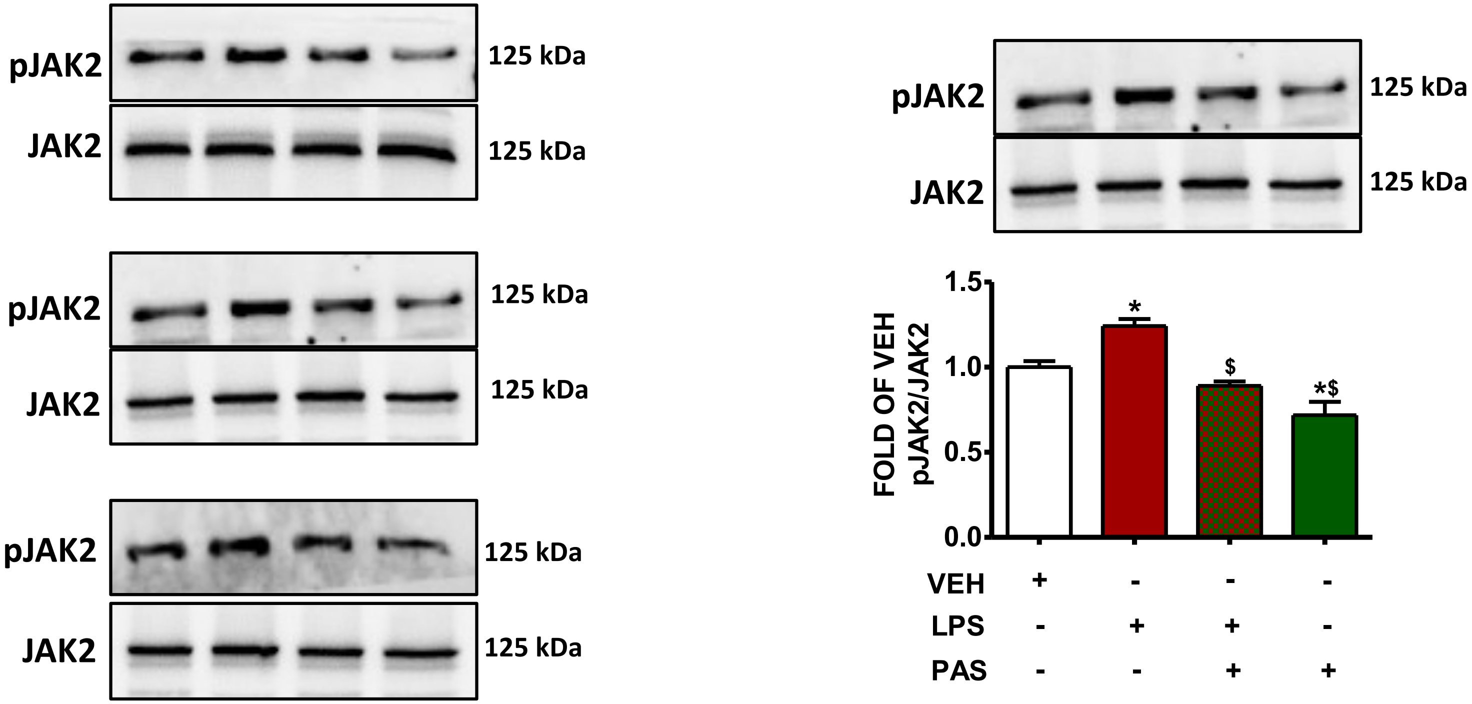
pJAK2
JAK2
125 kDa
FOLD OF VEH
pJAK2/JAK2
1.5
1.0
0.5
0.0
*
$
*$
VEH + - - -
LPS - + + -
PAS - - + +

Figure S1 (A)

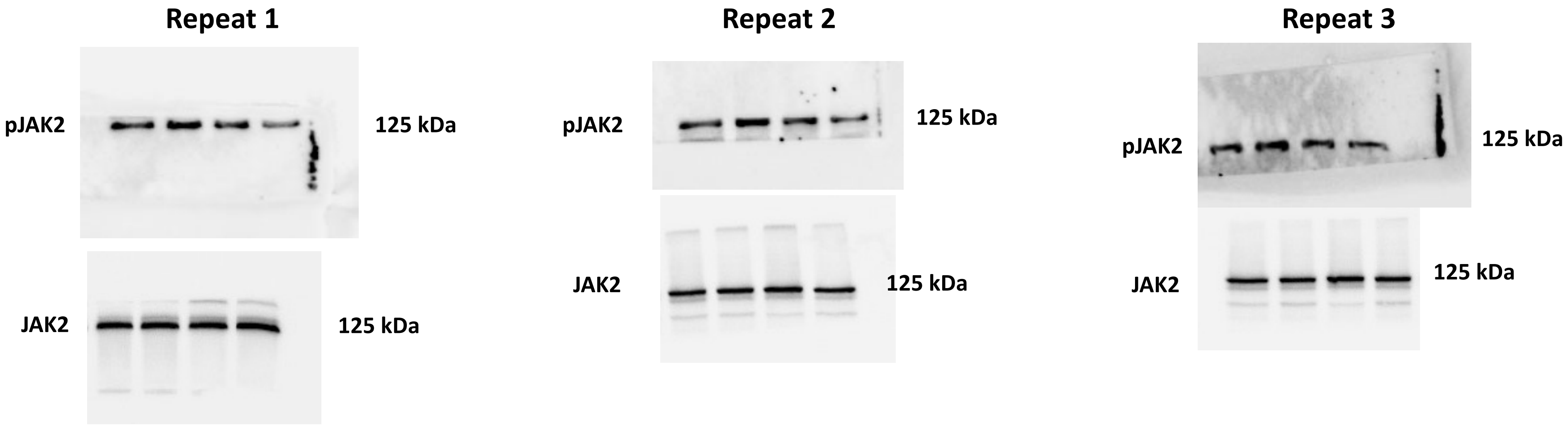
Repeat 1
pJAK2
125 kDa
JAK2
125 kDa
Repeat 2
pJAK2
125 kDa
JAK2
125 kDa
Repeat 3
pJAK2
125 kDa
JAK2
125 kDa

Figure S1 (B)

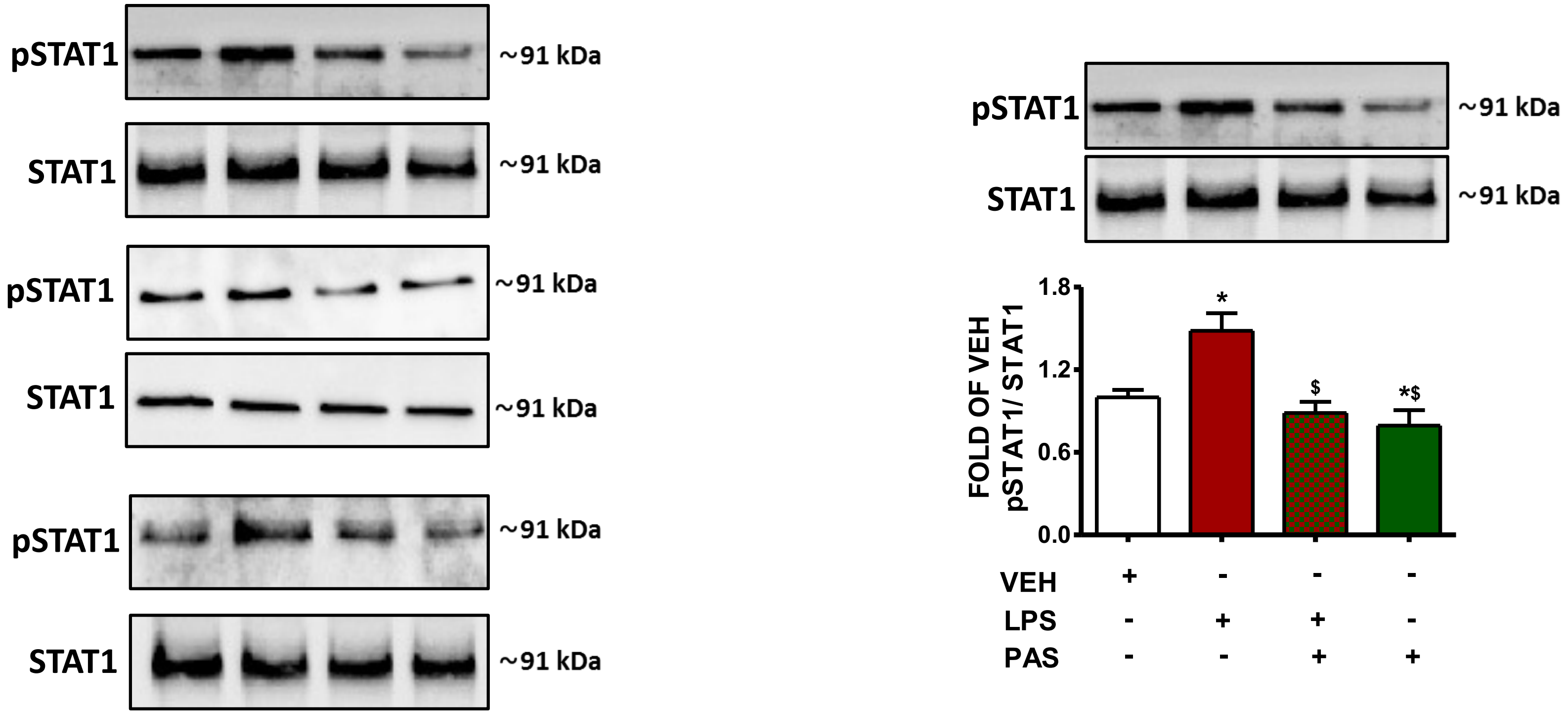

pSTAT1
~91 kDa
STAT1
~91 kDa
pSTAT1
~91 kDa
STAT1
~91 kDa
pSTAT1
~91 kDa
STAT1
~91 kDa
pSTAT1
~91 kDa
STAT1
~91 kDa
FOLD OF VEH
pSTAT1/ STAT1
1.8
1.2
0.6
0.0
*
$
*$
VEH + - - -
LPS - + + -
PAS - - + +

Figure S1 (B)

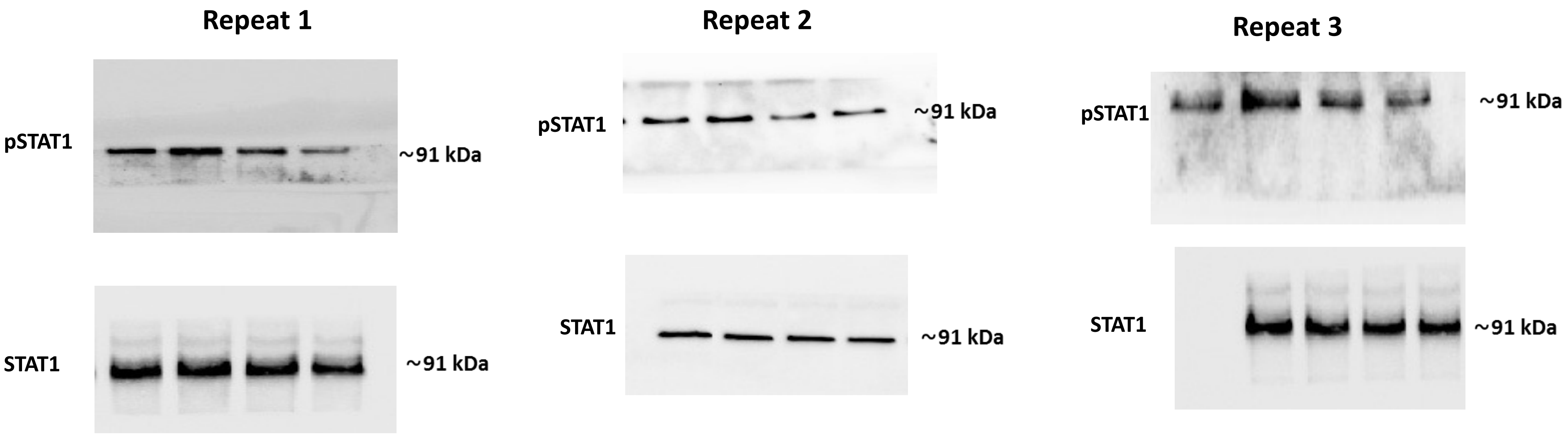
Repeat 1
pSTAT1
~91 kDa
STAT1
~91 kDa
Repeat 2
pSTAT1
~91 kDa
STAT1
~91 kDa
Repeat 3
pSTAT1
~91 kDa
STAT1
~91 kDa

Figure S1 (C)

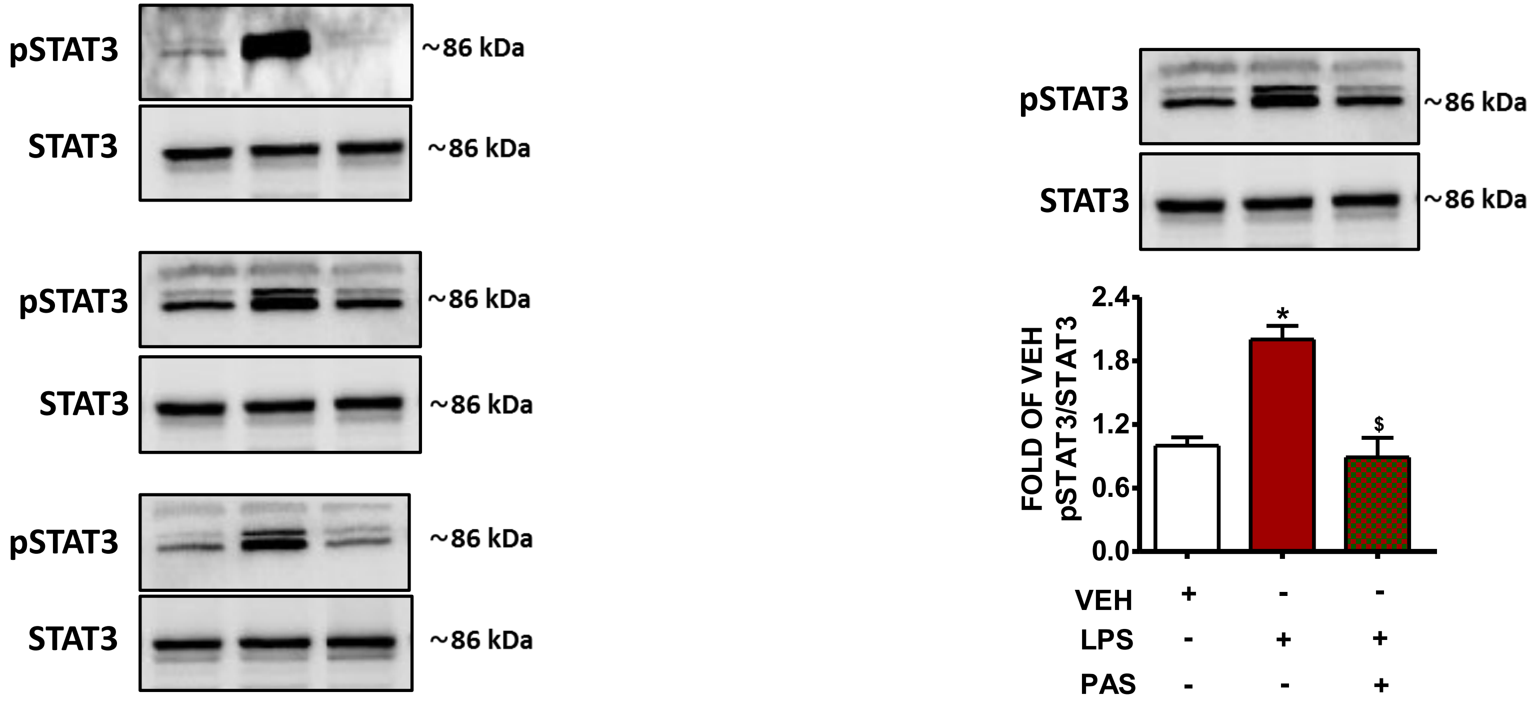
pSTAT3
~86 kDa
STAT3
~86 kDa
pSTAT3
~86 kDa
STAT3
~86 kDa
pSTAT3
~86 kDa
STAT3
~86 kDa
pSTAT3
~86 kDa
STAT3
~86 kDa
FOLD OF VEH
pSTAT3/STAT3
2.4
1.8
1.2
0.6
0.0
*
$
VEH + - -
LPS - + +
PAS - - +

Figure S1 (C)

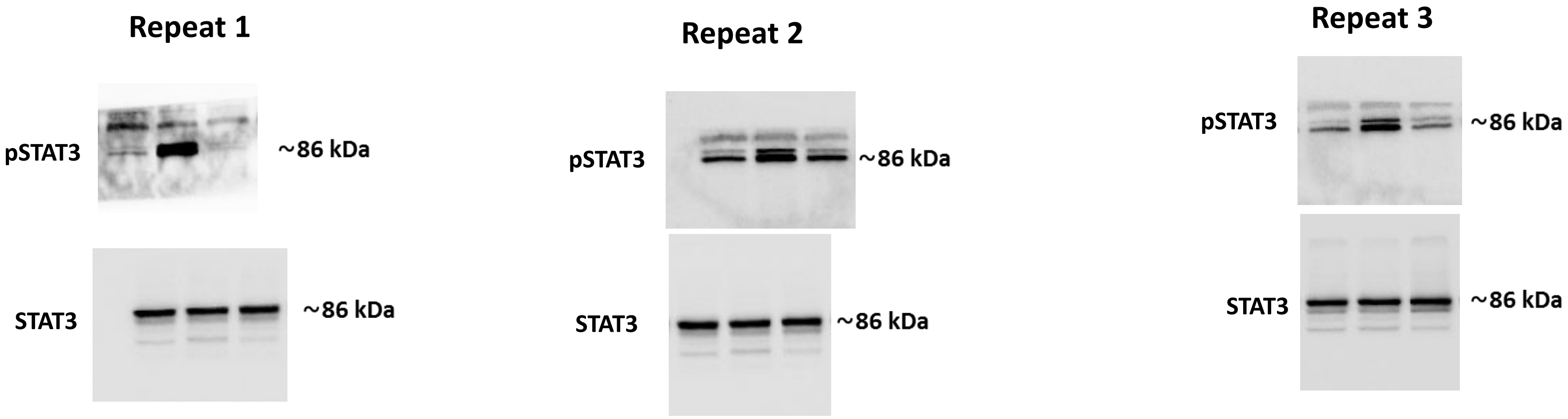

Repeat 1
pSTAT3
~86 kDa
STAT3
~86 kDa
Repeat 2
pSTAT3
~86 kDa
STAT3
~86 kDa
Repeat 3
pSTAT3
~86 kDa
STAT3
~86 kDa

Figure S2: original images of Figure 3

Effects of PAS in LPS-induced JNK, P38, and ERK1/2 phosphorylation

Figure S2 (A)

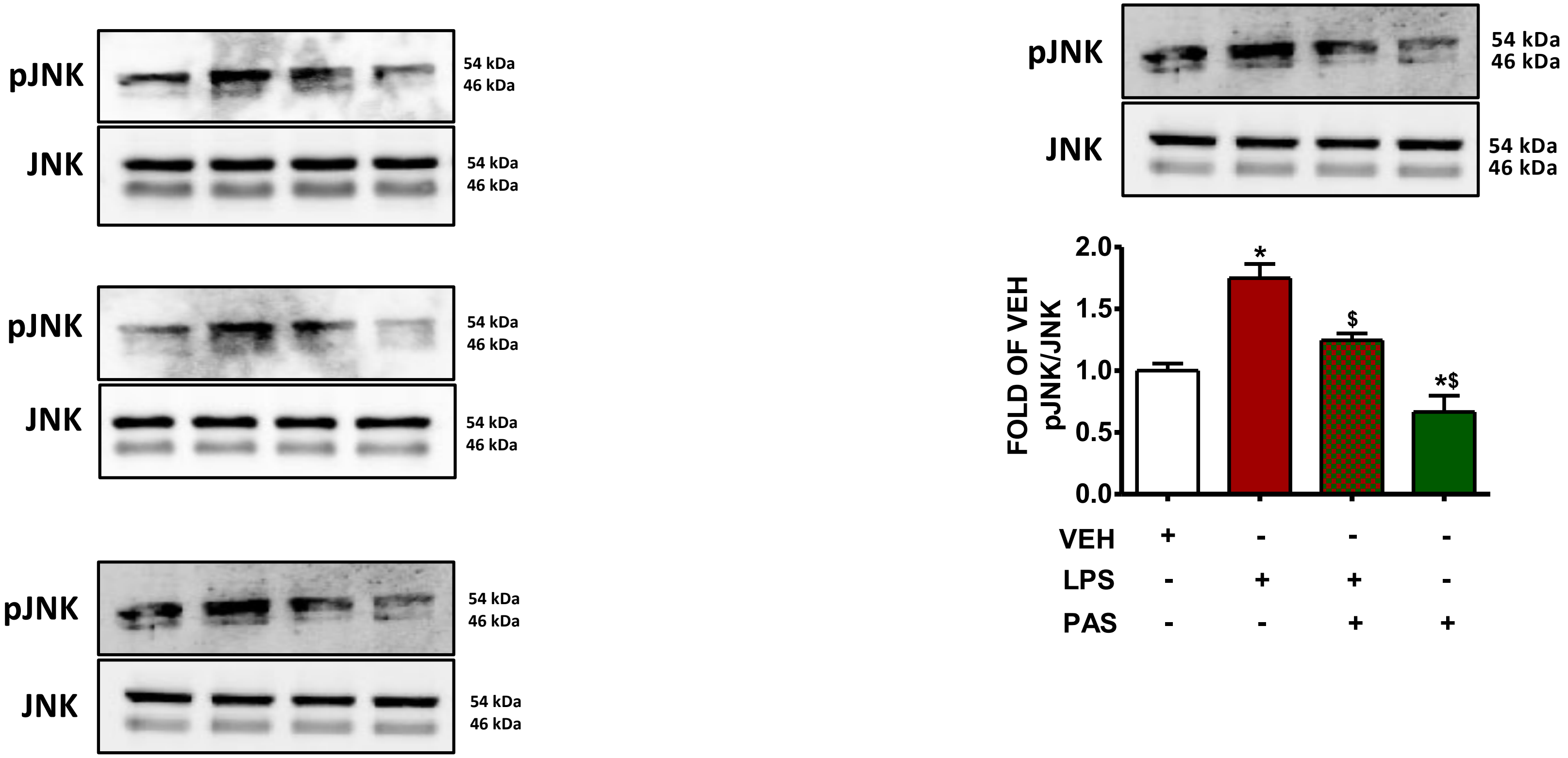
pJNK
54 kDa
46 kDa
JNK
54 kDa
46 kDa
pJNK
54 kDa
46 kDa
JNK
54 kDa
46 kDa
pJNK
54 kDa
46 kDa
JNK
54 kDa
46 kDa
pJNK
54 kDa
46 kDa
JNK
54 kDa
46 kDa
FOLD OF VEH
pJNK/JNK
2.0
1.5
1.0
0.5
0.0
*
$
*$
VEH + - - -
LPS - + + -
PAS - - + +

Figure S2 (A)

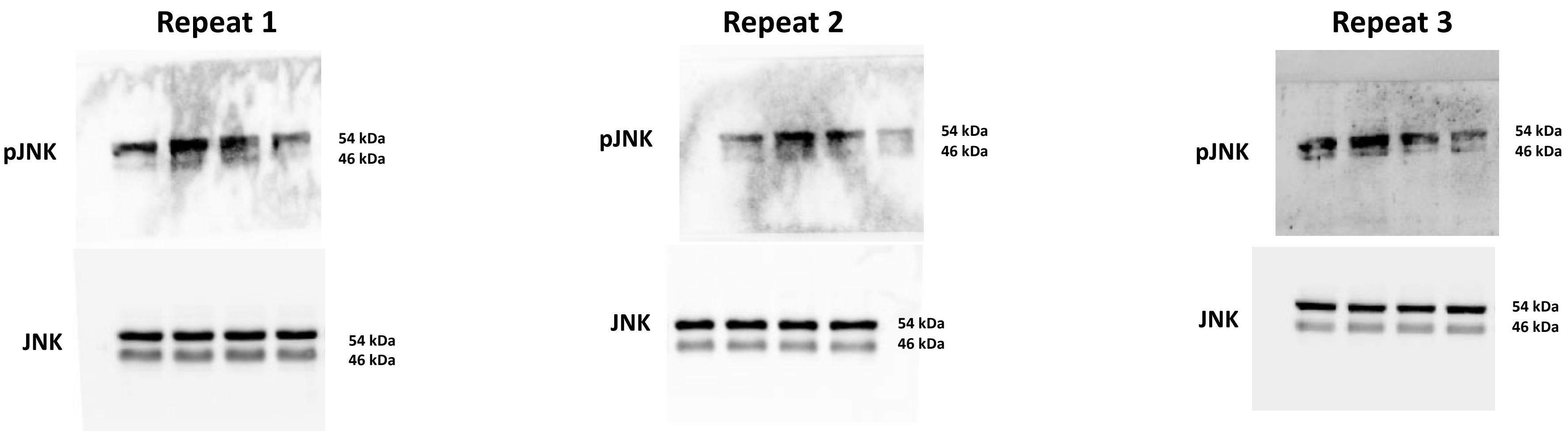

Repeat 1
pJNK
54 kDa
46 kDa
JNK
54 kDa
46 kDa
Repeat 2
pJNK
54 kDa
46 kDa
JNK
54 kDa
46 kDa
Repeat 3
pJNK
54 kDa
46 kDa
JNK
54 kDa
46 kDa

Figure S2 (B)

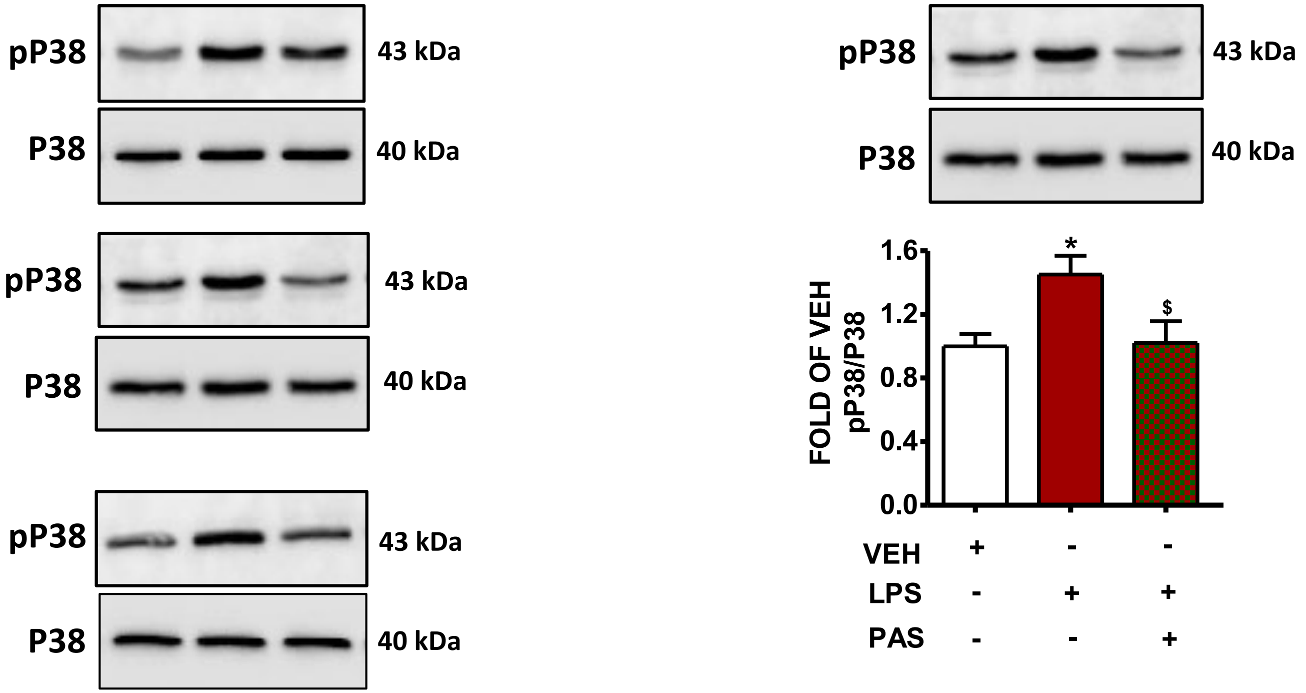
pP38
43 kDa
P38
40 kDa
pP38
43 kDa
P38
40 kDa
pP38
43 kDa
P38
40 kDa
pP38
43 kDa
P38
40 kDa
FOLD OF VEH
pP38/P38
1.6
1.2
0.8
0.4
0.0
*
$
VEH + - -
LPS - + +
PAS - - +

Figure S2 (B)

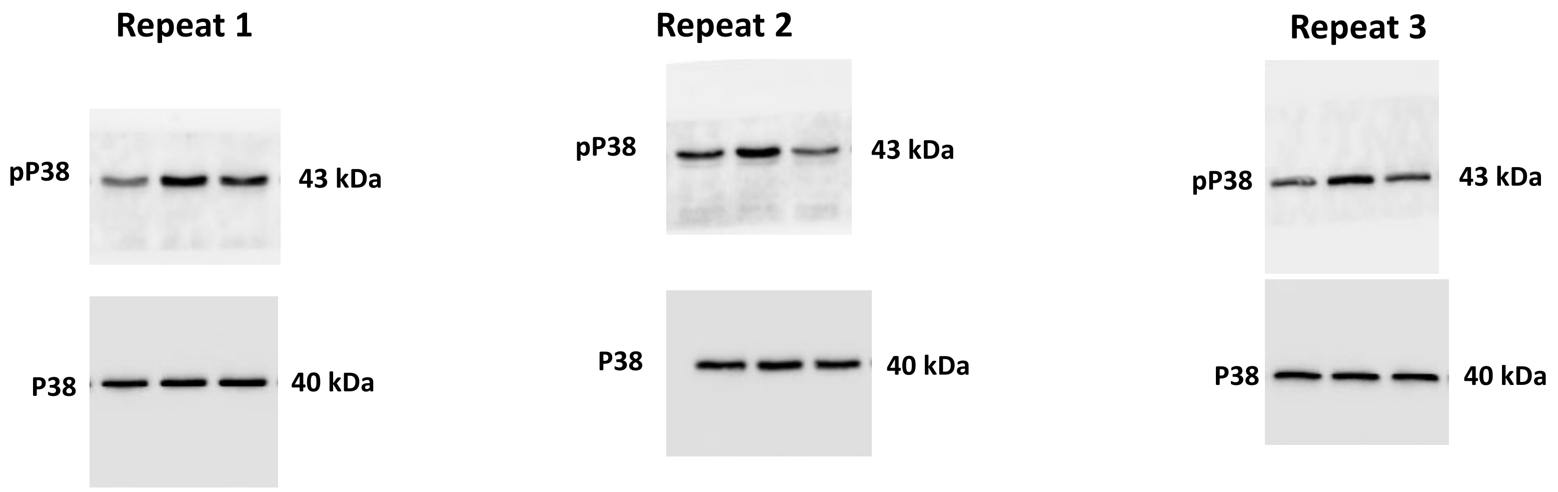
Repeat 1
pP38
43 kDa
P38
40 kDa
Repeat 2
pP38
43 kDa
P38
40 kDa
Repeat 3
pP38
43 kDa
P38
40 kDa

Figure S2 (C)

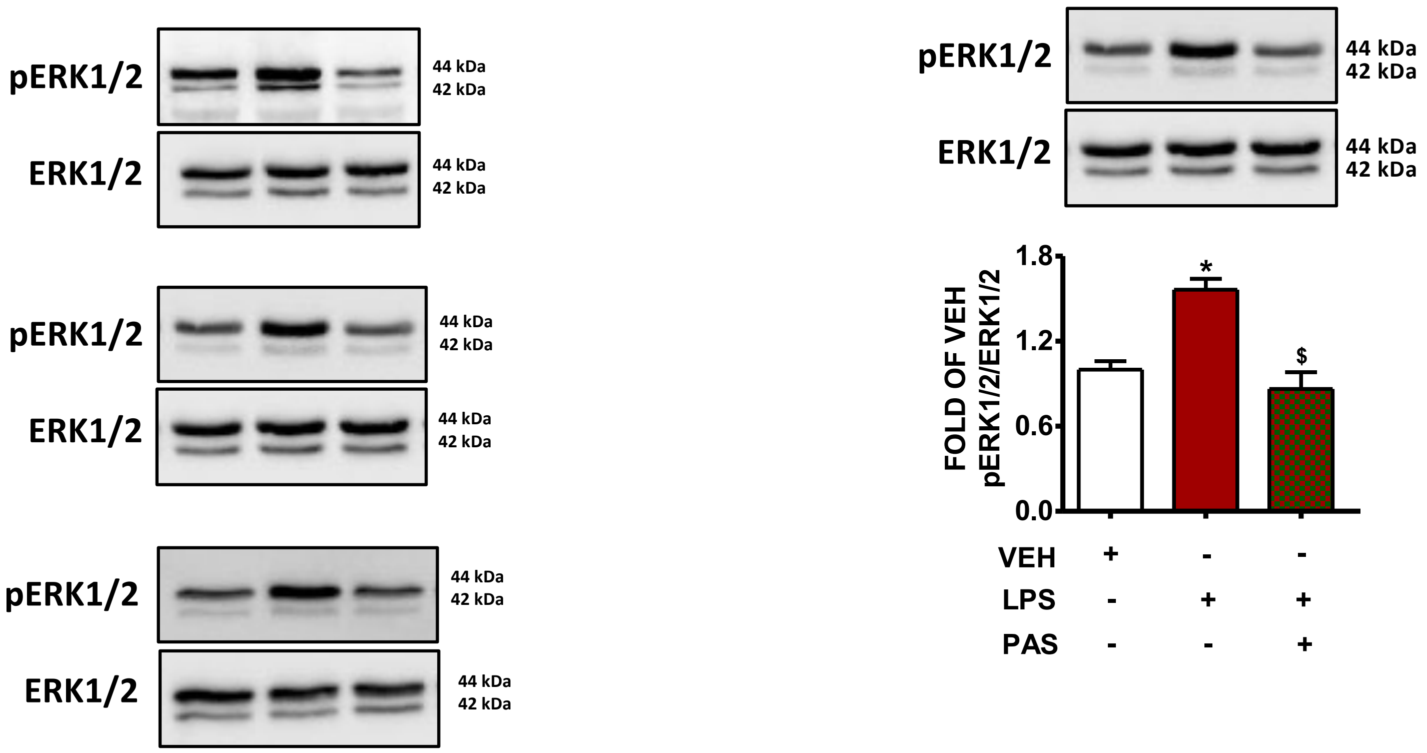

pERK1/2
44 kDa
42 kDa
ERK1/2
44 kDa
42 kDa
pERK1/2
44 kDa
42 kDa
ERK1/2
44 kDa
42 kDa
pERK1/2
44 kDa
42 kDa
ERK1/2
44 kDa
42 kDa
pERK1/2
44 kDa
42 kDa
ERK1/2
44 kDa
42 kDa
FOLD OF VEH
pERK1/2/ERK1/2
1.8
1.2
0.6
0.0
*
$
VEH + - -
LPS - + +
PAS - - +

Figure S2 (C)

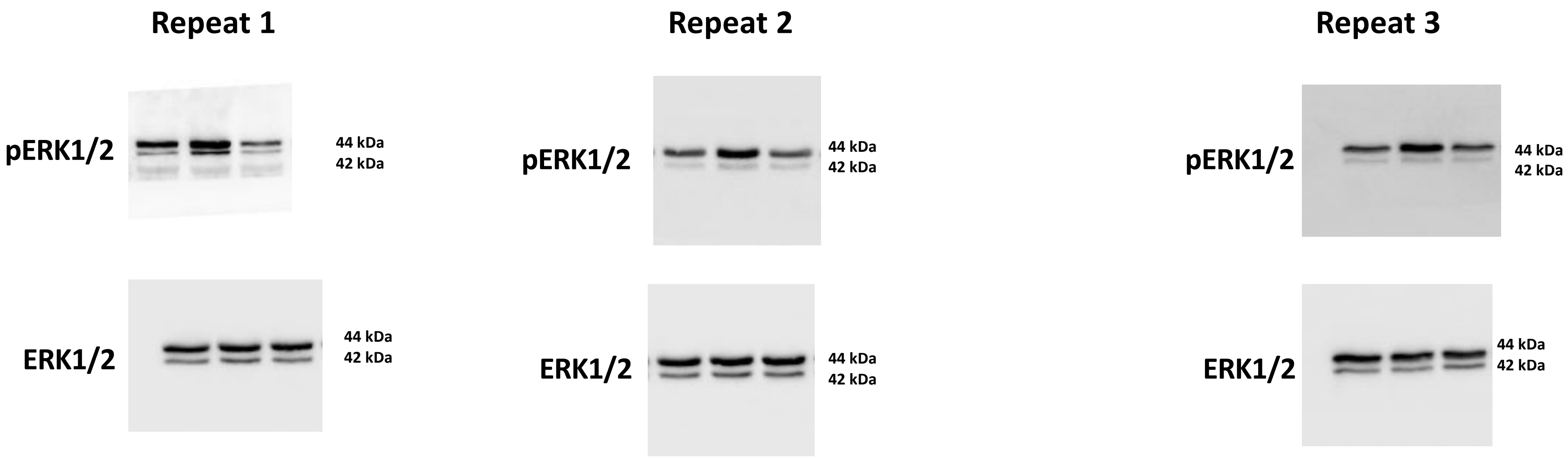
Repeat 1
Repeat 2
Repeat 3
pERK1/2
44 kDa
42 kDa
ERK1/2
44 kDa
42 kDa
pERK1/2
44 kDa
42 kDa
ERK1/2
44 kDa
42 kDa
pERK1/2
44 kDa
42 kDa
ERK1/2
44 kDa
42 kDa

Figure S3: original images of Figure 4

Effects of PAS in Grp94 expression

Figure S3

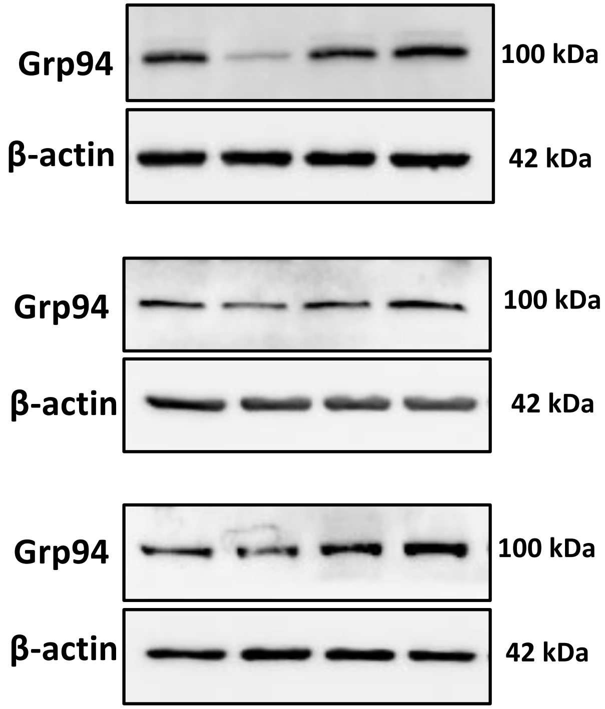
Grp94
100 kDa
β-actin
42 kDa
Grp94
100 kDa
β-actin
42 kDa
Grp94
100 kDa
β-actin
42 kDa

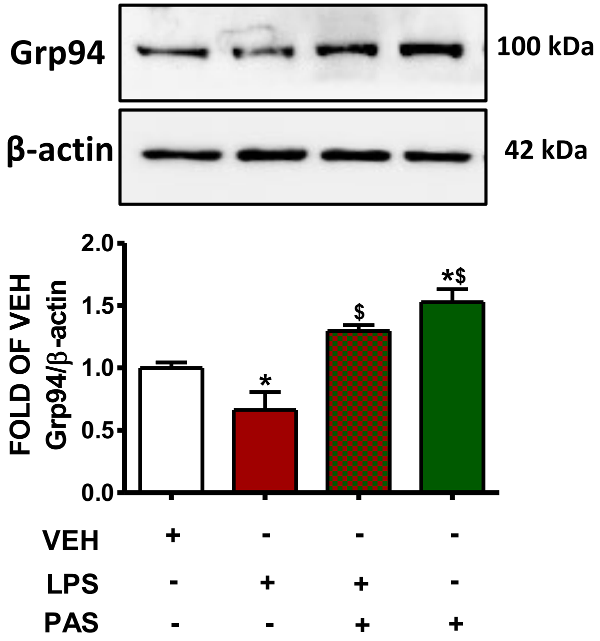
Grp94
100 kDa
β-actin
42 kDa
FOLD OF VEH
Grp94/β-actin
2.0
1.5
1.0
0.5
0.0
*
$
*$
VEH + - - -
LPS - + + -
PAS - - + +

Figure S3

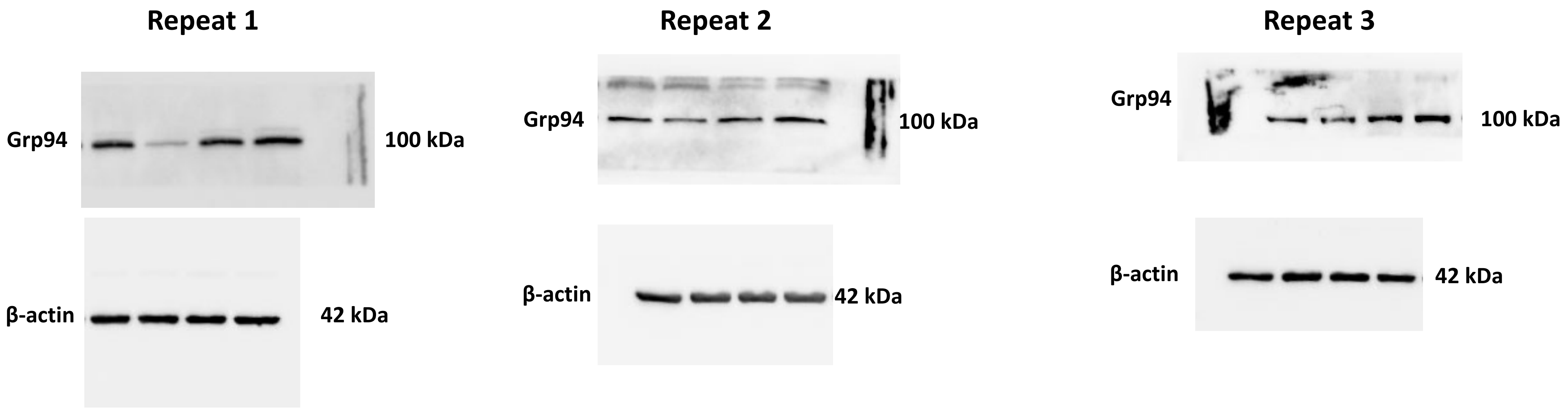
Repeat 1
Grp94
100 kDa
β-actin
42 kDa
Repeat 2
Grp94
100 kDa
β-actin
42 kDa
Repeat 3
Grp94
100 kDa
β-actin
42 kDa
